# Driver Characteristics Oriented Autonomous Longitudinal Driving System in Car-Following Situation

**DOI:** 10.3390/s20216376

**Published:** 2020-11-09

**Authors:** Haksu Kim, Kyunghan Min, Myoungho Sunwoo

**Affiliations:** 1 Department of Automotive Engineering, Hanyang University, Seoul 04763, Korea; yomovs@gmail.com; 2 Research & Development Division, Hyundai Motor Company, Hwaseong 18280, Korea; kyunghah.min@gmail.com

**Keywords:** personalized speed planning, autonomous longitudinal driving, individual driver behavior modeling, electric vehicle control

## Abstract

Advanced driver assistance system such as adaptive cruise control, traffic jam assistance, and collision warning has been developed to reduce the driving burden and increase driving comfort in the car-following situation. These systems provide automated longitudinal driving to ensure safety and driving performance to satisfy unspecified individuals. However, drivers can feel a sense of heterogeneity when autonomous longitudinal control is performed by a general speed planning algorithm. In order to solve heterogeneity, a speed planning algorithm that reflects individual driving behavior is required to guarantee harmony with the intention of the driver. In this paper, we proposed a personalized longitudinal driving system in a car-following situation, which mimics personal driving behavior. The system is structured by a multi-layer framework composed of a speed planner and driver parameter manager. The speed planner generates an optimal speed profile by parametric cost function and constraints that imply driver characteristics. Furthermore, driver parameters are determined by the driver parameter manager according to individual driving behavior based on real driving data. The proposed algorithm was validated through driving simulation. The results show that the proposed algorithm mimics the driving style of an actual driver while maintaining safety against collisions with the preceding vehicle.

## 1. Introduction

Automated vehicle control technologies such as advanced driver assistance system (ADAS) and autonomous vehicle (AV) have been researched over the years and have become the core of the future automotive industry [1,2]. The automated vehicle control fundamentally aims to provide comfort and safe driving for every user, which reduces the user’s burden of maneuvers and accidents caused by human error. Therefore, the ADAS is conventionally designed to guarantee general performance to satisfy unspecified individuals [3,4,5]. However, an assistance system that is optionally used by a specific user such as adaptive cruise control (ACC) must satisfy the user’s personal preference. Since the driver assistance technology only gets worth when it is actually employed, it should come up to the driver’s expectations regarding personal driving style and comfort. As a result, personalization of the ADAS has been researched to consider various personalities and to ensure a better user experience [6,7,8].

In this paper, we focus on the personalization of ACC that intervenes in longitudinal driving by controlling ego-vehicle speed and distance to a preceding vehicle in car-following situations. Car-following situations have become significantly frequent with the marked increase in traffic over the past several decades [9]. The ACC is a second generation of conventional cruise control (CCC) that helps drivers by taking on the role of longitudinal control of the car [10]. Since the CCC is effective in reducing the driver’s burden on the pedal operation and has the potential to improve the traffic flow, it has become a representative driver assistance system. The major difference between the ACC and the CCC is that the ACC system controls not only the ego-vehicle speed but also the spacing gap, which means the inter-vehicle distance from an ego-vehicle to a preceding vehicle. ACC utilizes radar technology to perceive the preceding vehicle’s motion and maintains the desired spacing gap to guarantee safety in car-following situations.

The importance of personalized ACC has long been recognized in the automotive industry, and researchers have conducted studies to reflect driver characteristics in conventional ACC to improve user acceptance. In several studies, heterogeneity of conventional ACC was confirmed through traffic simulation based on car-following models [11]. Car-following models were applied to represent specific driving behaviors. The study found that the performance of identical model differs by driver types; in other words, interpersonal variation affects more significantly than inter-model variation. 

Most previous studies for practical application of personalization proposed a set of predefined values by categorizing drivers into several groups [12,13,14]. For example, three predefined clusters of acceleration profile parameters were proposed. According to the driver, one of the three clusters is assigned to change the characteristics of the target acceleration profile. In addition, Rosenfeld et al. [13] classified drivers into the three groups through a large data set and predicted the Time Headway (THW) preferred by drivers in the group using a regression model and a decision tree. However, the group-based predefined sets of driving styles are difficult to reflect the great diversity of individual preferences and the fact that drivers might change their driving styles depending on driving conditions.

Therefore, several studies have proposed driver models that map vehicle states directly through deterministic equations into control inputs such as speed, acceleration, and pedal operation based on an individual’s driving style [15,16,17,18]. Wang et al. [15] designed a driver model that predicts pedal operation through an analytic equation based on THW and the inverse of Time-To-Collision (TTCi) based on a steady car-following data analysis. Butakov et al. [18] calibrated the parameters of the control policy to the driver for personalized ACC. They used a constant THW policy using a least-squares method to find the parameters of the THW model for three drivers. However, these approaches obtained the model parameters through offline learning based on a premeasured data set. 

In recent years, with the growth of computing devices and the rise of big data research, many researchers apply data-driven methods on driver behavior prediction. The Markov models stochastically predict driving states to inform the uncertainties of real-driving situations [19,20]. Besides, the deep neural network models [21,22,23] also have been popular because they can provide accurate prediction performance and handle a large amount of data. However, these methods are still unsuitable for use in real-time control systems, and online learning is difficult. 

The online update of personalization parameters is required to accommodate continuous changes in driver preferences [7]. In order to enable the online update of driving model parameters, Min et al. [24] proposed an online learning algorithm of model parameter structured in vector form. The online learning algorithm updates model parameters in real driving situations with short calculation time and validated in the real-time embedded system. However, this study was conducted in only vehicle deceleration conditions because the model parameters were utilized to predict the deceleration profile and applied to regenerative braking control [25].

In order to expand the previous research, we propose personalized ACC that is called Driver Characteristics Oriented ACC (DCO-ACC). The DCO-ACC is structured by a multi-layer framework composed of driver parameter manager, speed planner, and speed controller. The multi-layer framework is derived from the architecture of an autonomous driving system to ensure scalability and compatibility to AV or Connected Vehicle (CV) [26]. The driver parameter manager determines driver’s preferred driving feature as inputs for the speed planner according to current driving states. The speed planner generates an optimal speed profile reflecting individual driver characteristics with information from the parameter manager. The speed controller calculates target acceleration to track the planned speed profile. The core algorithm to mimic driver characteristics in the DCO-ACC is the driver parameter manager. The system manager models driving features reflecting individual driving styles based on real driving data. The driver parameter model uses the data-driven vector model and Gaussian distribution to probabilistically calculate driver parameters that change according to driving conditions. This method can flexibly cope with the uncertainty of actual operating conditions. The proposed longitudinal driving system was validated through simulation using IPG CarMaker, which provides a reliable environment for autonomous driving research. The simulation results were compared with actual driving data to show the similarity with the real driver behavior.

To clarify the contribution of this study, the two featured aspects of this study are summarized as follows. The first aspect is that the proposed driving system is structured based on the motion planning and control system of AVs. Since the previously proposed ACC personalization was based on conventional ACC structure, the controller components are dependent on each other, and compatibility for other control systems is not guaranteed. Therefore, the DCO-ACC is designed by combining the individual driver parameter management algorithm to a framework of longitudinal planning and control system of AVs. This multi-layer framework ensures compatibility with new additional functions and AV’s software structure. The second aspect is that the proposed driver parameter manager can learn online and have small-sized models. Previous driver models had bulky models that required large memories. Moreover, such models also should be updated offline with pre-logged data. However, since the proposed algorithm manages a driver parameter as a small-sized vector, it has advantages in terms of learning or memories.

The rest of this paper is arranged as follows. Section 2 introduces a real-driving data acquisition environment and analyzes individual driving behaviors in car-following situations. Section 3 briefly presents a system overview of the DCO-ACC and then describes each subsystem in detail. After that, the validation results through simulation studies are shown in Section 4, and the final section provides a conclusion.

## 2. Driver Characteristics in Car-Following Situation

### 2.1. Driving Data Acquisition

**Figure 1:**
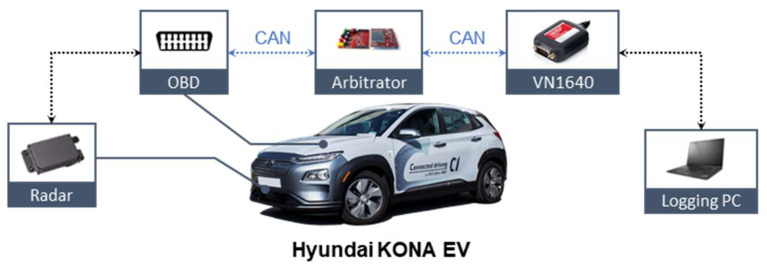
Vehicle configuration.

In order to design the longitudinal driving system reflecting individual driving styles, personal drivers’ driving data have been analyzed. The driving data for modeling of individual drivers were collected through real-driving tests with various in-vehicle sensors and radar, which has 150 m of maximum range, as shown in Table 1. Figure 1 shows an overall structure of the in-vehicle data flow. The data acquisition system was equipped with a target vehicle, Hyundai KONA EV. The in-vehicle sensors measure ego-vehicle driving states such as speed and acceleration. Besides, information on powertrain such as traction motor torque and motor speed also can be obtained. The radar provides the driving states of a preceding vehicle. It measures the spacing gap and the relative velocity between ego-vehicle and the preceding vehicle. The On-Board Diagnostics II (OBD II) was connected to the arbitrator using an Infineon TC237 through a Controller Area Network (CAN) protocol. The arbitrator parses and transfers the measured information to a logging system. The measured data were logged using Vector tools. Finally, the logging PC acquires and manages the CAN data. The measured data were logged using Vector tools. The real-time acquisition was synchronized and recorded at 100 Hz. 

**Figure 2:**
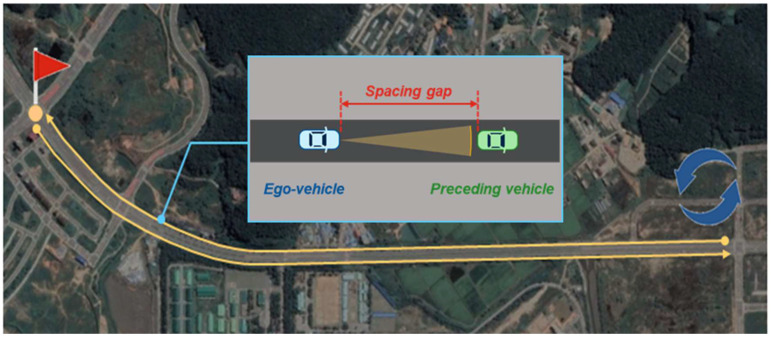
Driving data acquisition site.

A two-lane urban straight road in Incheon, Korea, was selected as a driving environment, as shown in Figure 2. The 2.0 km route represented by the yellow line in Figure 2 is the actual test route. Data acquisition experiments were conducted with three drivers in car-following situations. A total of 36 km of driving data were cumulated from the three round trips of the route by the three drivers who possess different styles of driving.

### 2.2. Driver Characteristics Analysis

Driver features in car-following situations are related to the ego-vehicle and the behavior of the preceding vehicle. Driver features related to the ego-vehicle include ego-vehicle velocity, acceleration, and jerk, i.e., a derivative of acceleration. Features related to the vehicle in front are spacing gap and relative velocity. Furthermore, THW and TTCi have been considered significant parameters to represent driver features. They are defined as in Equations (1) and (2), respectively:

(1) $$THW=\frac{d_{rel}}{v_{ego}},$$

(2) $$TTCi=\frac{d_{rel}}{v_{rel}},$$

where, $d_{rel}$ is the spacing gap, which is the distance between the preceding vehicle and ego-vehicle, $v_{rel}$ is the relative velocity, and $v_{ego}$ is the velocity of ego-vehicle.

Statistical analysis such as comparing the means and the standard deviations of these driver features to distinguish the driving styles of individual drivers in car-following situations has been used in several studies [12,14,27]. Furthermore, many researchers have speculated that the distribution of driver features, especially THW, is a significant factor for distinguishing individual drivers in car-following situations. The achievements of these previous studies by the driving data obtained in the previously mentioned acquisition environment were confirmed.

**Figure 3:**
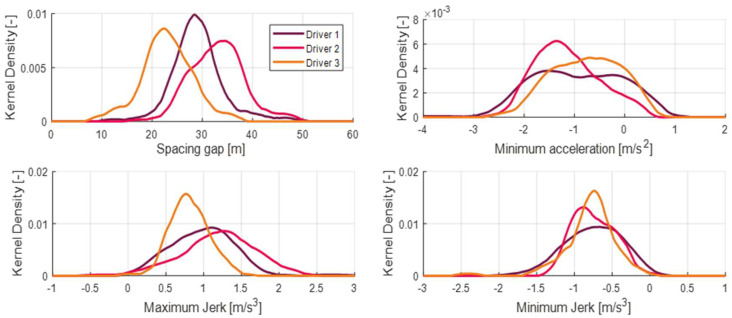
Kernel density estimation (KDE) of driver features; spacing gap, maximum jerk, and minimum jerk.

In this paper, Kernel Density Estimation (KDE) was utilized to analyze driving features differed by drivers. The KDE is a statistical approach to estimate the probability density of datasets difficult to be expressed by parametric density functions. In fact, driving datasets show irregular patterns caused by protean real-driving situations. As shown in Figure 3, the driving data construct different kernel density according to the driver. Typically, the probability density of the spacing gap shows a clear difference by each driver. The difference of spacing gap distribution indicates the fact that each driver prefers a different safe distance to the preceding vehicle. Additionally, the jerk, gradient of ego-vehicle’s acceleration, also differs by each driver.

In addition, jerk is the index that best represents the feeling of deceleration felt by the driver, and the maximum and minimum values of this jerk show different distributions for each driver. Therefore, if you design a longitudinal controller that follows the vehicle ahead by reflecting the distance to the vehicle in front, the jerk’s maximum and minimum values, this can satisfy individual drivers.

**Figure 4:**
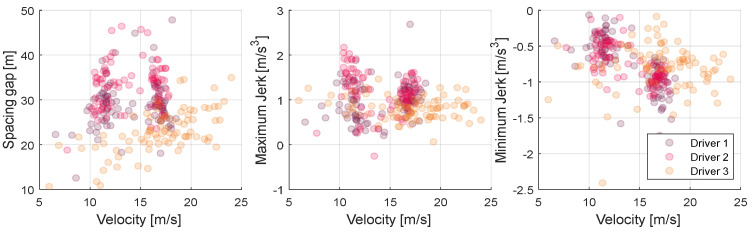
Driving feature distributions according to ego-vehicle velocity; distributions of spacing gap, maximum jerk and minimum jerk.

Furthermore, each of the analyzed driver features not only appears differently for each driver but also appears differently depending on the current driving condition of the vehicle. In other words, the driver feature is not defined as one value for one driver but should be modeled differently for one driver for each situation. Figure 4 is a graph showing the trend of the three driver features introduced above according to the ego-vehicle speed. For example, the orange driver in the first orange graph maintains a short spacing gap overall compared to other drivers while moderately increasing the distance between cars as the vehicle speed increases. However, in the case of magenta drivers, they stick to the distance between vehicles within a certain range regardless of the vehicle speed. Therefore, to design a driving system that effectively reflects the driver’s propensity, it is necessary to design a model capable of estimating the driver feature that changes according to the driving state in real-time. The method of modeling the characteristics within the proposed algorithm is described in the next section. 

## 3. Driver Characteristics Oriented Autonomous Longitudinal Driving System

### 3.1. System Overview

**Figure 5:**
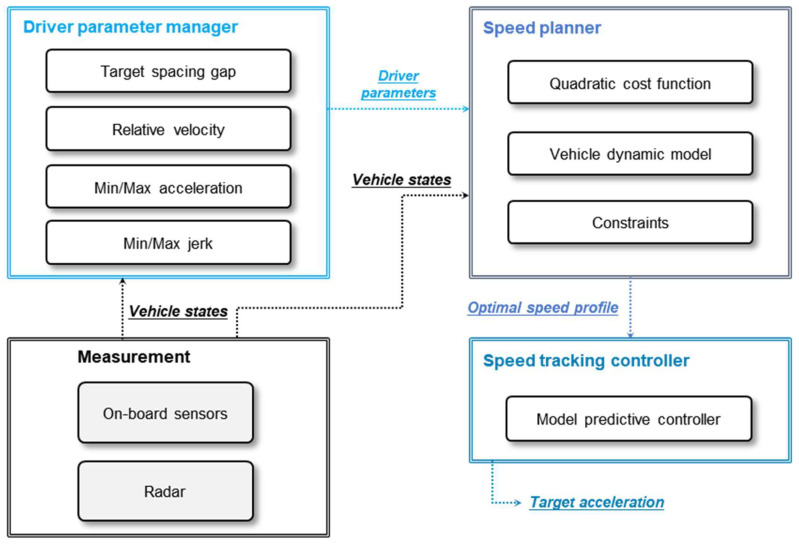
The overall framework of driver characteristics oriented adaptive cruise control (DCO-ACC).

The proposed longitudinal driving system calculates the desired acceleration according to driving conditions and individual driver’s characteristics. As shown in Figure 5, the proposed algorithm consists of three subsystems: driver parameter manager, speed planner, and speed tracking controller. At first, the driver parameter manager determines the target spacing gap for each individual driver. The target spacing gap was modeled by each driver’s driving data to reflect the driver’s characteristics. After that, the speed planner generates an optimal speed profile to keep the target spacing gap between ego-vehicle and the preceding vehicle. In this process, the speed planner considers the dynamics of the two vehicles and system constraints based on the Quadratic Programming (QP) to guarantee safety and drivability. At last, the speed tracking controller calculates the desired acceleration to follow the optimal speed profile based on the Model Predictive Controller (MPC). The speed controller makes it a top priority to follow the speed profile.

### 3.2. Driver Parameter Manager

The driver parameter manager decides the driver’s preferred spacing gap that has been modeled by individual driving data in a car-following situation. According to the driving data, the spacing gap is a representative feature that varies depending on the driver. This is because the driver maintains a safe distance to the preceding vehicle to avoid a collision, and the distance each driver feels safe is different. In addition, the proposed parameter manager calculates maximum and minimum values of acceleration and jerk. These values indicate the feeling of acceleration that drivers can endure. For example, some drivers may be afraid of excessive acceleration. Conversely, other drivers may be bored with too low acceleration. The driver parameter manager determines parameter values based on real driving data to reflect such different preferences of each driver.

**Figure 6:**
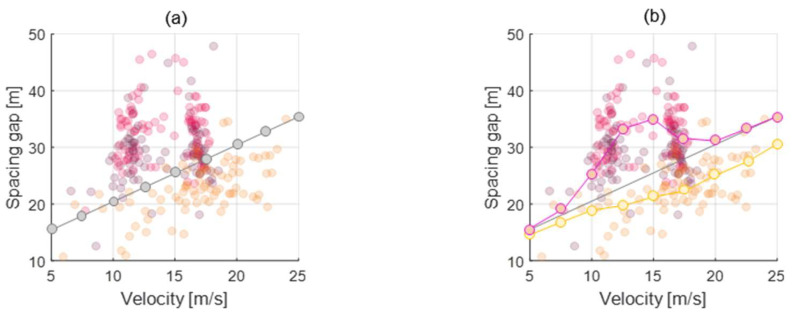
Parameter vector of spacing gap depending on the ego-vehicle velocity. (**a**) Initial parameter vector, (**b**) Updated parameter vector.

In order to manage the driver parameters, parameter values are defined as the vector array value according to the index parameter. The index parameter represents a driving state related to the driver parameter. For example, the spacing gap, one of the driver parameters, is highly correlated to the ego-vehicle velocity for all drivers, as shown in Figure 6a. This parameter represents that drivers desire further spacing gap to ensure driving safety as velocity increases. Furthermore, each driver shows a different spacing gap preference. Therefore, the parameter manager can determine the appropriate driver parameter value according to the vehicle state. In Figure 6a, the initial parameter vector is derived as a gray dot line that represents the average driving style for every driver. After that, the parameter manager updates the initial parameter vector using the real-driving data of personal drivers. As a result, the update algorithm learns and reflects the individual driver’s driving style, as shown by two kinds of dot lines in Figure 6b.

The parameter vector is derived from the driving data analyzed in Section 2. First, the initial vector is generated using the entire data. After that, data are added for each driver, and the initial vector is updated according to the difference between the actual data and the initial vector, and the driver parameter vector can be obtained. This process proceeds with the following equations. The update target value $\delta_{\theta }$ is determined using the activated value $\theta_{act}$ and reference value $\theta_{ref}$ of each parameter with a learning rate α as Equation (3). The activated value is a driver parameter according to the current parameter vector, and the reference value is a value from newly updated data. After that, the update target value $\delta_{\theta }$ is used to update the driver parameter vector $V_{\theta }$ as Equation (4).

(3) $$\delta_{\theta }=\alpha \left(\theta_{ref}-\theta_{act}\right),$$

(4) $$V_{\theta }{\left(i\right)}^{+}=V_{\theta \;}{\left(i\right)}^{-}+\psi \left(i\right)\delta_{\theta },$$

**Figure 7:**
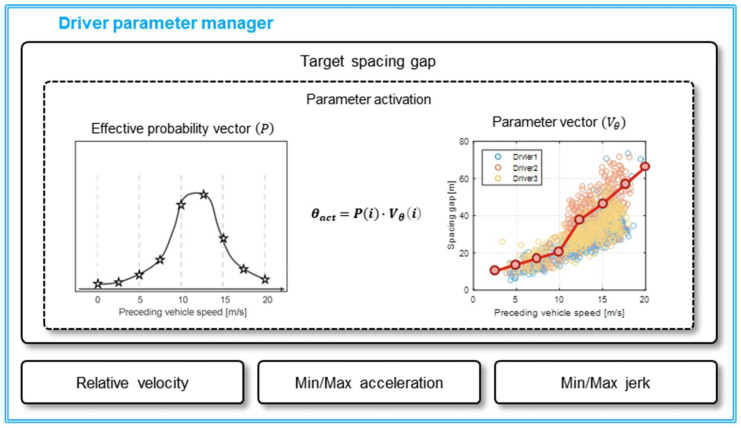
Parameter activation of driver parameter manager.

**Figure 8:**
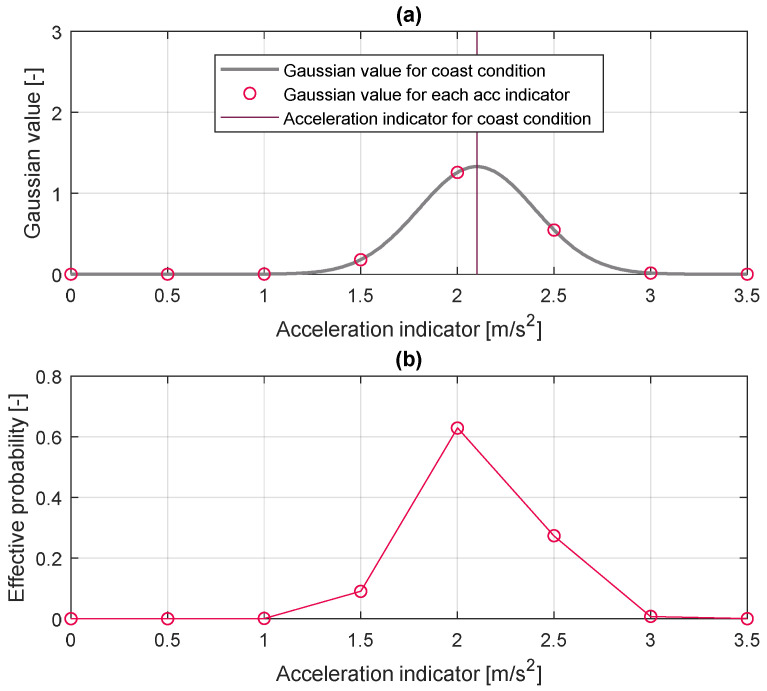
Gaussian value and effective likelihood according to acceleration indicator value. (**a**) Gaussian value, (**b**) Effective likelihood.

Based on the parameter vectors and an effective probability vector, the parameter activation algorithm calculates the driver parameters. Figure 7 shows the process of activating the driver parameter based on data. The trained driver parameter vector is conducted dot product with the effective probability vector while the algorithm is running to calculate the final driver parameter. The effective probability vector is derived by sampling at the same resolution as the driver parameter from the Gaussian distribution. As mentioned earlier, the index parameter is correlated to the parameter vector and represents some driving states. The currently measured index value is set as the mean value, and the predefined standard deviation is used. It produces a Gaussian distribution as shown in Figure 8. The effective probability is determined by normalizing this Gaussian distribution for each index value as Equation (5).

(5) $$P\left(i\right)=Norm\left(\frac{1}{\sigma \sqrt{2\pi }}e^{{\left(\frac{i-i_{val}}{2\sigma }\right)}^{2}}\right),$$

where the P(i) is an effective probability for each vector index i, $i_{val}$ is the index value for the current driving state, and σ is the standard deviation of the index value. For example, when the index parameter is determined as 2.1 value according to the driving state as shown in Figure 8a, the effective probability is also determined based on the Gaussian distribution, as shown in Figure 8b. The effective probability of indicator 2 is the largest value because the index value of the current driving state is 2.1 value. Then, the proper parameter value for current driving status is activated by the inner product of the effective probability vector and the driver parameter vector as Equation (6).

(6) $$\theta_{act}=P\left(i\right)\cdot V_{\theta }\left(i\right),$$

where $\theta_{act}$ is an activated parameter and $V_{\theta }\left(i\right)$ is vector value for parameter θ.

### 3.3. Speed Planner

The speed planner produces the optimal speed profile to control the spacing gap between ego-vehicle and preceding vehicle because the spacing gap should be controlled according to the driver’s preferred value to reflect the driver’s personality. Additionally, the speed profile must be able to drive safely in a real vehicle.

**Figure 9:**
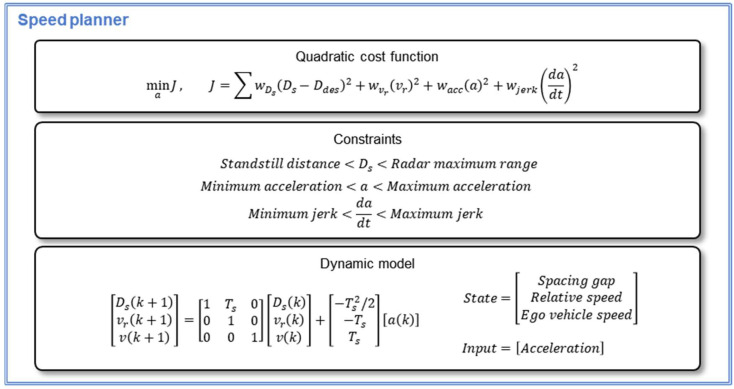
Optimization problem definition of the speed planner.

Therefore, the generation of the speed profile is defined as an optimization problem that is composed of vehicle dynamics and system constraints, as shown in Figure 9. The QP was applied to solve the optimization problem by minimizing a quadratic cost function of Equation (7).

(7) $$J=\sum w_{D_{s}}{\left(D_{s}-D_{des}\right)}^{2}+w_{v_{r}}{\left(v_{r}\right)}^{2}+w_{acc}{\left(a\right)}^{2}+w_{jerk}{\left(\frac{da}{dt}\right)}^{2},$$

where $D_{s}$ is a measured spacing gap, $D_{des}$ is target spacing gap from the parameter manager, $v_{r}$ is relative velocity, v is ego-vehicle speed, a is ego-vehicle acceleration as a control input, and $\frac{da}{dt}$ is the jerk. Four kinds of ω are weights for each term in the cost function. Since the first component of the cost function is the spacing gap error, minimization of the cost function means minimization of spacing gap error.

(8) $$\left[\begin{array}{c} D_{s}\left(k+1\right)\\ \begin{array}{c} v_{r}\left(k+1\right)\\ v\left(k+1\right) \end{array} \end{array}\right]=\left[\begin{array}{ccc} 1 & T_{s} & 0\\ 0 & 1 & 0\\ 0 & 0 & 1 \end{array}\right]\left[\begin{array}{c} D_{s}\left(k\right)\\ \begin{array}{c} v_{r}\left(k\right)\\ v\left(k\right) \end{array} \end{array}\right]+\left[\begin{array}{c} -T_{s}^{2}/2\\ \begin{array}{c} -T_{s}\\ T_{s} \end{array} \end{array}\right]\left[a\left(k\right)\right],$$

In addition, the vehicle dynamics model of Equation (8) was applied to estimate driving states and calculate the cost function in every control period. The state vector contains the spacing gap, relative velocity, and ego-vehicle speed. Ego-vehicle acceleration takes the role of control input. 

Furthermore, the proposed speed planner considers the system constraints, as shown in Figure 9. According to the vehicle specification and driving comfort, acceleration and jerk of the ego-vehicle should be constrained. For example, the spacing gap is constrained according to safe standstill distance and a measurable range of radar specifications. The constraints are also determined by individual drivers’ parameters. For example, jerk is limited by the maximum and minimum jerk calculated for each driver in the driver parameter manager in the optimization process. 

### 3.4. Speed Tracking Controller

The speed tracking controller calculates the target acceleration required for the vehicle to follow the planned speed profile. Since the lower-level controller of an autonomous driving system of EV generally calculates the desired motor torque to follow the target acceleration, the target acceleration must be calculated. The speed tracking controller was designed by Model Predictive Controller (MPC). The optimal speed profile is used as a reference input. A simple velocity model is used as the dynamics model, and the acceleration *a*(*t*) serves as a control input.

(9) V(t+1)=V(t)+a(t)dt,

The controller is only aiming at tracking performance for the planned speed profile. This is because the speed planner considered both driver propensity and vehicle dynamics. Therefore, a cost function consists of only the error between the target speed profile and the estimated speed profile. The MPC searches for the acceleration value that minimizes the cost function of speed error, which means that speed tracking performance has the highest priority. Besides, only the minimum limiting conditions are applied to the acceleration value. The acceleration value is constrained based on the range that the vehicle can achieve within its own capabilities. 

## 4. Validation

### 4.1. Simulation Environment

In order to validate the proposed DCO-ACC, a simulation environment including the test track, preceding vehicle, and radar sensor was developed with vehicle simulation software, IPG CarMaker; this provides the various sensor models required for ADAS and is a simulation software that can freely configure the shape of test track and the behavior of the surrounding vehicles. In addition, it has detailed fidelity at the vehicle powertrain level and has been used extensively in the research of AVs and CVs, as well as ADAS. We modeled the Hyundai KONA EV to build a simulation environment similar to the data acquisition environment. Table 2 shows the specifications of the target vehicle.

Furthermore, the speed tracking controller was implemented to control the vehicle in IPG CarMaker to generate output from the proposed speed planner. It takes the role of a higher-level controller as a Model Predictive Control (MPC)-based controller that can systematically handle the nonlinearities and constraints of the vehicle system. Based on the target acceleration from the speed tracking controller, a lower-level controller as a simple Proportional-Integral-Derivative (PID)controller outputs the normalized pedal position.

Besides, the proposed algorithm and IPG CarMaker were integrated through “generic.mdl”, which is the Simulink model supported by IPG CarMaker to verify the proposed planning algorithm designed with MATLAB/Simulink. In the generic model, based on the IPG CarMaker for Simulink (CM4SL) library, internal signals such as the accelerator and brake pedal position could be modified, and the control output of the velocity tracking controller such as the accelerator and brake pedal positions were connected to the internal variables of the virtual vehicle. The entire simulation environment was constructed. First of all, in the MATLAB/Simulink, the proposed planning algorithm and velocity tracking controller were implemented, and the normalized pedal position was output, as described previously. Secondly, the CM4SL library was used to convert the control output to the CarMaker variables in the generic model. Third, the ego-vehicle was controlled via the input pedal position in the IPG CarMaker. Finally, the CarMaker variables, including the ego-vehicle states and the preceding vehicle states, were fed back into the proposed algorithm and speed tracking controller.

The vehicle in car-following situations was simulated with the proposed driving system using the different driver parameters for the identical preceding vehicle behavior of premeasured driving data in Section 2. The aim was to verify whether the proposed algorithm was similar to the driver by comparing the driving results with the human manual driving data. For example, when the preceding vehicle data of 1st driver’s actual data were replayed as a simulation input, the personalized driving system adaptively used the driver parameters modeled by 1st driver’s driving data. The driver parameters were the target spacing gap, minimum and maximum values of acceleration and jerk, and relative velocity, as shown in Figure 5. The driver parameters affect the speed planner to generate a speed profile reflecting individual driving behaviors. Then, the personalized driving system is simulated to drive the modeled target vehicle to follow the preceding vehicle that moves exactly like the vehicle that the 1st driver was actually following. If the proposed algorithm mimics the driver well, it will drive while maintaining the speed and spacing gap similar to the actual driver. 

### 4.2. Simulation Results

#### 4.2.1. Personalized Longitudinal Driving Results

**Figure 10:**
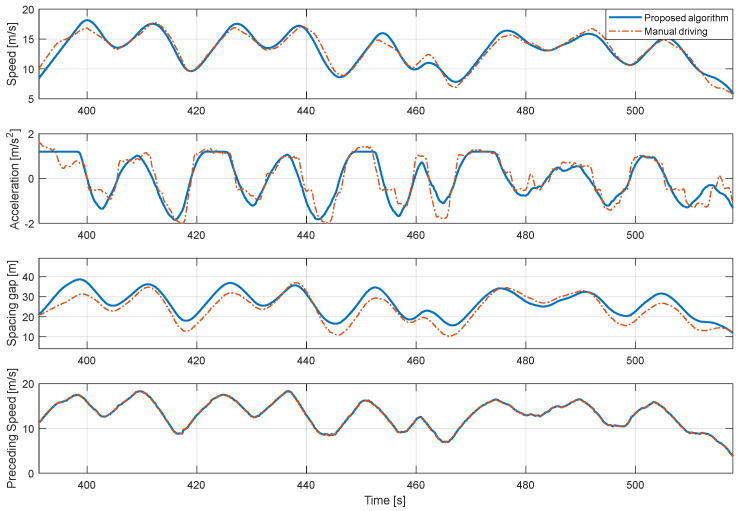
Comparison of 1st driver’s real-driving data and simulation results of the proposed algorithm with 1st driver model.

Figure 10 is a graph comparing the simulation results of the proposed algorithm for the motion of the same preceding vehicle with the actual driving result of the 1st driver. Therefore, the preceding vehicle speed profile appears the same. The results of the speed profile and spacing gap show that the driving result of the proposed algorithm effectively mimics the actual driving result of the 1st driver. Referring to the acceleration graph, the results of the proposed algorithm has less variation compared to the actual driving data. Acceleration in actual driving contains a lot of sudden changes because it contains sensor noise and is sensitive to the driver’s pedal operation, which contains unpredictably sudden change. On the other hand, in the case of the proposed algorithm result, since the target velocity profile is generated by preventing a sudden change in acceleration during the optimization process in the speed planner, the velocity and acceleration results appear more stable than the actual one. 

Furthermore, algorithms for each driver and actual driving results were compared based on Root Mean Square Error (RMSE) for vehicle speed and distance between vehicles in Table 3. The quantitative results show that the distinguished performance of driving behavior replication. The target spacing gap effectively mimics the actual driving data so that the driving speed is similar to the actual drivers’ behavior.

**Figure 11:**
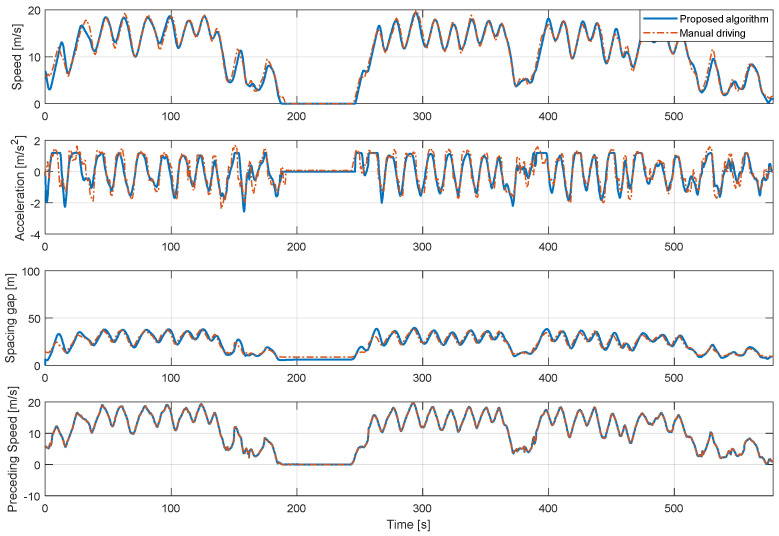
Driving simulation results of the proposed algorithm of 1st driver for the entire route.

**Figure 12:**
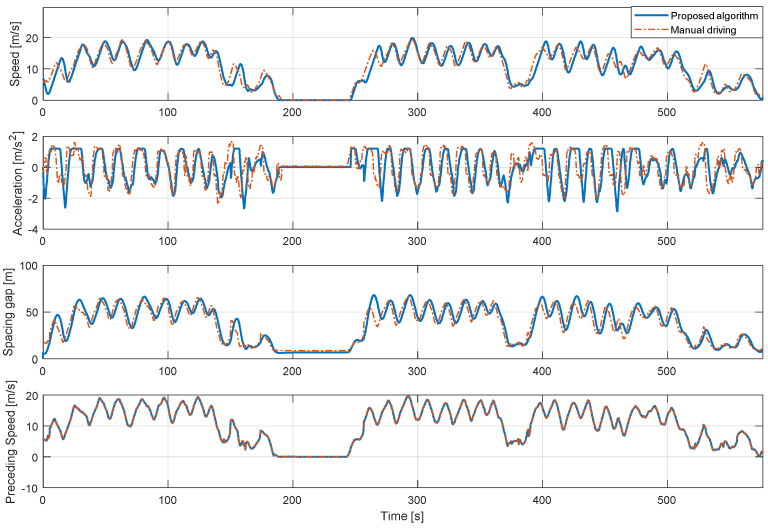
Driving simulation results of the proposed algorithm of 2nd driver for the entire route.

**Figure 13:**
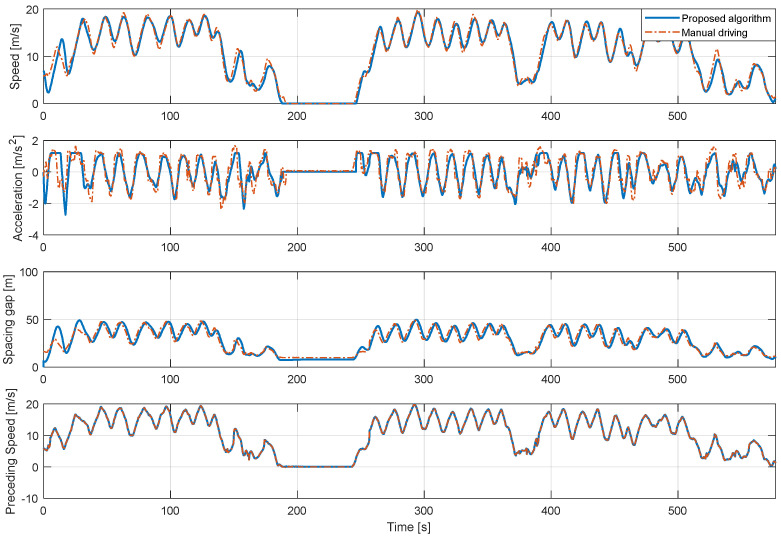
Driving simulation results of the proposed algorithm of 3rd driver for the entire route.

In addition, Figure 11, Figure 12 and Figure 13 show the longitudinal driving results of the real-driving data for each driver and the proposed algorithm for each driver model in the entire route. The driver model about dynamic conditions was validated by proving ground driving and real road driving. Real road driving contains various urban and highway driving environments. In the proving ground results, the vehicle driving occurred by the motion of a preceding vehicle. The results shown in Figure 13 are consistent in the stable acceleration and deceleration as the preceding vehicle drove intentionally. The control results show also that the proposed algorithm predicts the speed profile at each driving case through the preceding vehicle’s effect as well. The model did not predict the deceleration on some deceleration cases because the vehicle deceleration was not caused by the preceding vehicle.

#### 4.2.2. Effect of Variety of Driver Parameters

**Figure 14:**
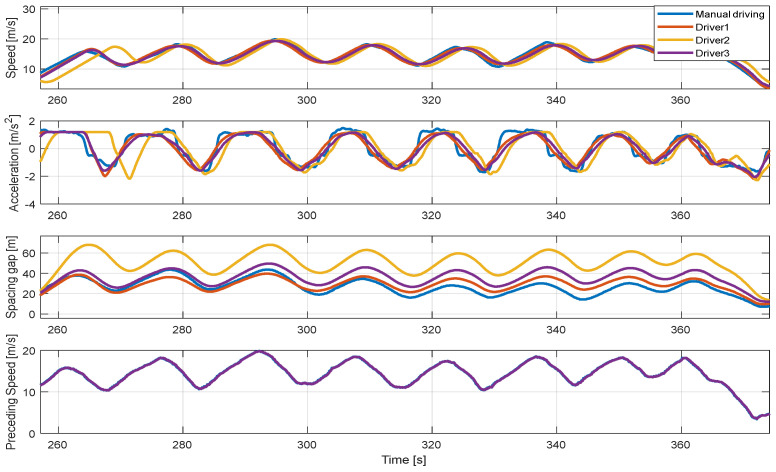
Driving behaviors of each driver model for identical preceding vehicle’s behavior.

The simulation results show that for the same preceding vehicle, only the ego-vehicle behavior differed according to the driver model used in the proposed algorithm, as shown in Figure 14. These results show the driving results of the proposed algorithm using different driver parameters when the initial conditions of driving states are constrained to be the same. For example, the 2nd driver has the characteristic of maintaining a safe distance farther than other drivers in real-driving data. As such, the proposed algorithm with the 2nd driver model maintains a large safety distance compared to other driver models. In particular, the spacing gap trajectories show that the proposed planning algorithm controls the vehicle while maintaining the spacing gap that actual drivers prefer. 

#### 4.2.3. Statistical Validation

**Figure 15:**
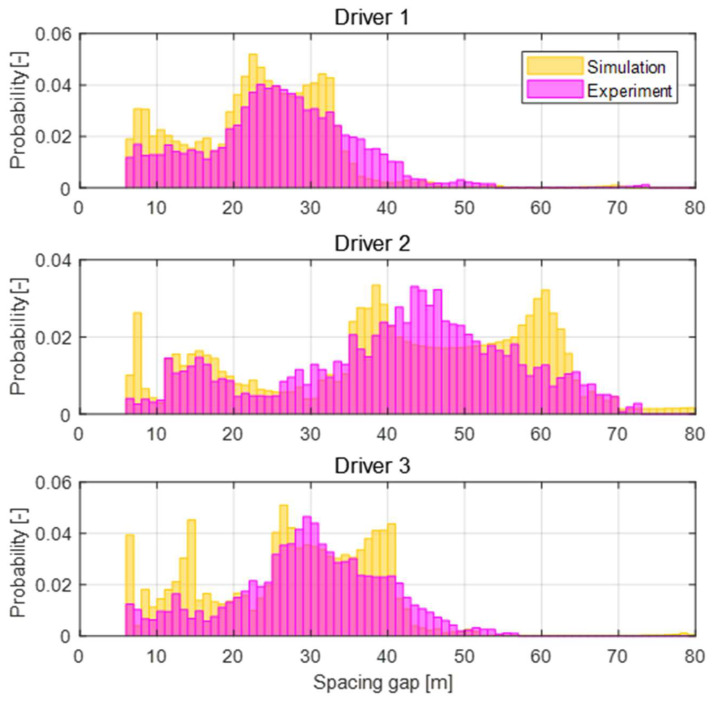
Spacing gap distributions of driving data and proposed algorithm results by each driver.

The K-S distance and the K-L divergence were used to objectively evaluate the similarity between the driving style of the proposed planning algorithm and the human manual driving style. The K-S distance and the K-L divergence are indicators that can measure the similarity between two distributions and are respectively defined as in the following equations [28]:

(10) $$D_{KS}=\underset{x}{\max}\left|F_{1}\left(x\right)-F_{2}\left(x\right)\right|,$$

(11) $$D_{KL}(P_{1}||P_{2})=-\Sigma P_{1}\left(x\right)\log\left(\frac{P_{2}\left(x\right)}{P_{1}\left(x\right)}\right),$$

where $P_{1}\left(x\right)$ and $P_{2}\left(x\right)$ denote the Probability Density Functions (PDF) of x and $F_{1}\left(x\right)$ and $F_{2}\left(x\right)$ denote the Cumulative probability Density Functions (CDF) of x for the two different distributions. The K-S distance can compute the maximum differences in the shape and the location of the PDF, while the K-L divergence can calculate the sum of the differences at each index x of the two PDFs. The similarity between the driving style of the personalized driving system and human manual driving is validated by the similarity of the spacing gap distributions, as shown in Figure 15 because the proposed speed planner aims to mimic the spacing gap behaviors. The K-S distance and K-L divergence between the two distributions for the spacing gap are summarized in Table 4. The values for all drivers are lower than 0.2, which implies a high similarity between the two distributions. As a result, the proposed system was validated to mimic actual driving behaviors.

## 5. Conclusions

In this paper, we proposed the driver characteristics oriented autonomous longitudinal driving system in the car-following situation. The proposed driving system was constructed by three subsystems: driver parameter manager, speed planner, and speed tracking controller. First, the driver parameter manager calculates driving parameters that are defined through driving feature analysis based on real-driving data. The driver parameters represent the personal driver behaviors in the car-following situation. Then, the speed planner generates the target speed profile through the optimization process. In order to make the speed planner special, the optimization problem contains the driver parameter to reflect individual driver characteristics. Finally, the speed tracking controller determines to follow the target speed profile. Since the structure of the proposed driving system is based on a general autonomous driving system, it can be easily expanded to autonomous driving. The proposed algorithm was validated through simulations. Since the optimization parameters are determined based on individual driving behaviors, the proposed driving system reduces the sense of heterogeneity for a specific driver. 

In future research, the proposed algorithm will be applied to the autonomous driving system in real-driving situations. The operating condition should be extended from car-following situations to various driving situations such as traffic jam or cut-in situations. Accordingly, the driver parameter manager will be modified to handle additional driving parameters practically. Besides, the target vehicle will be equipped with a motor torque control interface. The motor torque control interface is necessary to access the motor control unit and intervene control process. Furthermore, the speed tracking controller of the proposed driving system can be advanced to reflect the EVs’ powertrain characteristics. The MPC of the tracking controller will be modified to consider motor-to-wheel dynamics to compensate for the influence of vehicle inertia and improve speed tracking performance.

## Figures and Tables

**Table 1:** Radar specification.

Index	Value
Maximum range	150 m
FOV (Field of View)	+/− 10 degrees over 60 m +/− 45 degrees under 60 m
Update rate	50 ms

**Table 2:** Specification of the target vehicle (Hyundai KONA EV).

Vehicle Parameter	Value	Powertrain Parameter	Value
Unloaded weight	1685 kg	Maximum torque (Motor)	395 Nm
Length	4180 mm	Maximum power (Motor)	150 kW
Width	1800 mm	Maximum rpm (Motor)	11,000 rpm
Height	1570 mm	Inertia (Motor)	0.028 kg·m^2^
Driving axle	Front driven	Capacity (Battery)	180 Ah 64 kWh
Tire specification	215/55 R	Idle voltage (Battery)	353 V
Tire radius	17 inch	Maximum power (Battery)	150 kW

**Table 3:** Error of velocity and spacing gap by drivers.

Driver #	RMSE of Speed [m/s]	RMSE of Spacing Gap [m]
1	1.1101	6.6511
2	2.4716	15.6221
3	1.2282	9.5933
Average	1.6033	10.6222

**Table 4:** K-S distance and K-L divergence values of the spacing gap distributions.

Driver #	K-S Distance	K-L Divergence
1	0.1605	0.1365
2	0.1887	0.1602
3	0.1726	0.1402
Average	0.1739	0.1456

## References

[1] Bishop R. A survey of intelligent vehicle applications worldwide. Proceedings of the IEEE Intelligent Vehicles Symposium 2000 (Cat. No. 00TH8511).

[2] Xiao L., Gao F. (2010). A comprehensive review of the development of adaptive cruise control systems. Veh. Syst. Dyn..

[3] González D., Pérez J., Milanés V., Nashashibi F. (2016). A Review of Motion Planning Techniques for Automated Vehicles. IEEE Trans. Intell. Transp. Syst..

[4] Paden B., Čáp M., Yong S.Z., Yershov D., Frazzoli E. (2016). A Survey of Motion Planning and Control Techniques for Self-Driving Urban Vehicles. IEEE Trans. Intell. Veh..

[5] Bianco C.G.L., Piazzi A., Romano M. Velocity planning for autonomous vehicles. Proceedings of the IEEE Intelligent Vehicles Symposium.

[6] Dey K.C., Yan L., Wang X., Wang Y., Shen H., Chowdhury M., Yu L., Qiu C., Soundararaj V. (2016). A Review of Communication, Driver Characteristics, and Controls Aspects of Cooperative Adaptive Cruise Control (CACC). IEEE Trans. Intell. Transp. Syst..

[7] Hasenjäger M., Wersing H. Personalization in advanced driver assistance systems and autonomous vehicles: A review. Proceedings of the 2017 IEEE 20th International Conference on Intelligent Transportation Systems (ITSC).

[8] Martinez C.M., Heucke M., Wang F.Y., Gao B., Cao D. (2018). Driving Style Recognition for Intelligent Vehicle Control and Advanced Driver Assistance: A Survey. IEEE Trans. Intell. Transp. Syst..

[9] Brackstone M., McDonald M. (1999). Car-following: A historical review. Transp. Res. Part F Traffic Psychol. Behav..

[10] Rajamani R. (2012). Vehicle Dynamics and Control.

[11] Ranjitkar P., Nakatsuji T., Kawamua A. (2005). Car-Following Models: An Experiment Based Benchmarking. J. East. Asia Soc. Transp. Stud..

[12] de Gelder E., Cara I., Uittenbogaard J., Kroon L., van Iersel S., Hogema J. Towards personalised automated driving: Prediction of preferred ACC behaviour based on manual driving. Proceedings of the 2016 IEEE Intelligent Vehicles Symposium (IV).

[13] Rosenfeld A., Bareket Z., Goldman C.V., LeBlanc D.J., Tsimhoni O. (2015). Learning Drivers’ Behavior to Improve Adaptive Cruise Control. J. Intell. Transp. Syst..

[14] Zhu B., Jiang Y., Zhao J., He R., Bian N., Deng W. (2019). Typical-driving-style-oriented Personalized Adaptive Cruise Control design based on human driving data. Transp. Res. Part C Emerg. Technol..

[15] Wang J., Zhang L., Zhang D., Li K. (2013). An Adaptive Longitudinal Driving Assistance System Based on Driver Characteristics. IEEE Trans. Intell. Transp. Syst..

[16] Mikami K., Okuda H., Taguchi S., Tazaki Y., Suzuki T. Model predictive assisting control of vehicle following task based on driver model. Proceedings of the 2010 IEEE International Conference on Control Applications.

[17] Liebner M., Baumann M., Klanner F., Stiller C. Driver intent inference at urban intersections using the intelligent driver model. Proceedings of the 2012 IEEE Intelligent Vehicles Symposium.

[18] Butakov V., Ioannou P. Driving Autopilot with Personalization Feature for Improved Safety and Comfort. Proceedings of the 2015 IEEE 18th International Conference on Intelligent Transportation Systems.

[19] Kuge N., Yamamura T., Shimoyama O., Liu A. (2000). A Driver Behavior Recognition Method Based on a Driver Model Framework. SAE Trans..

[20] Oliver N., Pentland A.P. (2000). Driver Behavior Recognition and Prediction in a Smart Car. Proc. SPIE Int. Soc. Opt. Eng..

[21] Morton J., Wheeler T.A., Kochenderfer M.J. (2017). Analysis of Recurrent Neural Networks for Probabilistic Modeling of Driver Behavior. IEEE Trans. Intell. Transp. Syst..

[22] Yu H., Wu Z., Wang S., Wang Y., Ma X. (2017). Spatiotemporal Recurrent Convolutional Networks for Traffic Prediction in Transportation Networks. Sensors.

[23] Huang X., Sun J., Sun J. (2018). A car-following model considering asymmetric driving behavior based on long short-term memory neural networks. Transp. Res. Part C Emerg. Technol..

[24] Min K., Sim G., Ahn S., Sunwoo M., Jo K. (2019). Vehicle Deceleration Prediction Model to Reflect Individual Driver Characteristics by Online Parameter Learning for Autonomous Regenerative Braking of Electric Vehicles. Sensors.

[25] Sim G., Min K., Ahn S., Sunwoo M., Jo K. (2019). Deceleration Planning Algorithm Based on Classified Multi-Layer Perceptron Models for Smart Regenerative Braking of EV in Diverse Deceleration Conditions. Sensors.

[26] Jo K., Kim J., Kim D., Jang C., Sunwoo M. (2014). Development of Autonomous Car—Part I: Distributed System Architecture and Development Process. IEEE Trans. Ind. Electron..

[27] Moon S., Yi K. (2008). Human driving data-based design of a vehicle adaptive cruise control algorithm. Veh. Syst. Dyn..

[28] Lefèvre S., Carvalho A., Borrelli F. (2016). A Learning-Based Framework for Velocity Control in Autonomous Driving. IEEE Trans. Autom. Sci. Eng..

